# Study of Electrospun Membranes Composed of PCL and Tilapia-Skin Collagen with Tetracycline or Chloramphenicol in Contact with Human Skin Fibroblasts for Wound Dressing Treatment

**DOI:** 10.1021/acsomega.3c06354

**Published:** 2024-01-24

**Authors:** Abraham Alejandro Leyva-Verduzco, Jesús Manuel Quiroz Castillo, Lerma Hanaiy Chan-Chan, Claudia Georgina Ramirez-Mendoza, María Mónica Castillo Ortega, Damián Francisco Plascencia-Martínez, Itzel Yanira López-Peña, Celia Olivia García-Sifuentes

**Affiliations:** † Departamento de Investigación en Polímeros y Materiales, Universidad de Sonora, 83000 Hermosillo Sonora, Mexico; ‡ Departamento de Física, Universidad de Sonora, 83000 Hermosillo Sonora, Mexico; § Centro de Investigación en Alimentación y Desarrollo A. C., Coordinación Tecnología de alimentos de Origen Vegetal, 83304 Hermosillo Sonora, Mexico; ∥ Laboratorio de Bioquímica y Calidad de Productos Pesqueros, Centro de Investigación en Alimentación y Desarrollo A. C., Coordinación Tecnología de alimentos de Origen Animal, 83304 Hermosillo Sonora, Mexico

## Abstract

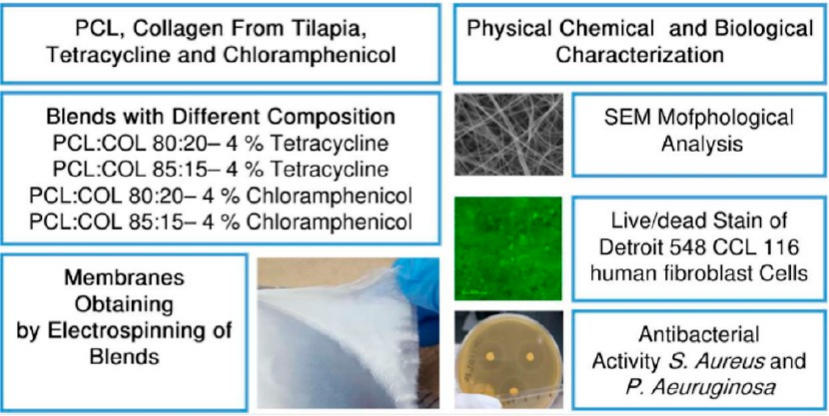

Diabetic foot ulcers are a common complication of diabetes mellitus and can lead to severe infections and delayed wound healing. The development of effective wound dressings is crucial to promoting faster healing and preventing infections. This investigation aims to fabricate and characterize electrospun meshes composed of poly(ε-caprolactone) and collagen, extracted from tilapia skin. Additionally, tetracycline and chloramphenicol were incorporated into the dressings to explore their potential to combat wound infections. A comprehensive characterization was carried out, covering the physical structure, chemical composition, and potential application-related properties of the materials by the combination of scanning electron microscopy, Fourier transform infrared (FTIR), mechanical analysis, cell viability, live/dead staining, and microbiological analysis. Changes in mechanical properties were observed, related to the morphology of the membranes; the presence of the active molecules is evidenced by FTIR analysis; cell viability above control was observed for all the prepared membranes, and they were active in antimicrobial tests, suggesting that the developed materials have the potential to be further explored as wound dressings or scaffolds for diabetic foot ulcers.

## Introduction

Diabetes is a chronic metabolic disease characterized by high glucose levels in the blood. It is accompanied by modifications in the metabolism of carbohydrates, lipids, and proteins. Chronic hyperglycemia is associated with long-term complications in the eyes, kidneys, nerves, blood vessels, and heart.^1^ In addition, foot ulcers are a common problem in people with diabetes. Ulcers are accompanied by neuropathy, peripheral blood vessel disease and infection, and may culminate in gangrene, causing limb amputation.^2^ It is estimated that 15% of the people who suffer from diabetes will develop foot ulcers, therefore, the mechanisms to treat them are of great importance.^3,4^ An approach to care for these injuries is the use of drug-delivery dressings that boost the healing process and promote antimicrobial activity.^5^

Dressings for refractory wounds are commonly composed of fibrous membranes such as those produced by electrospinning techniques. This system can generate fibers with diameters ranging from nano- to microscale that can mimic the extracellular matrix (ECM) and act as a scaffold where different types of cells can grow.^6^ Furthermore, electrospinning is expected to remain a popular nanotechnology in laboratories and expand its presence in industrial production. The growing demand for nanomaterials, ongoing research and development efforts, advancements in equipment, and the technology’s adaptability to various applications ensure its continued relevance and growth in the foreseeable future in industry.^7^

Synthetic materials such as polystyrene (PS), poly(vinyl alcohol) (PVA), polyglycolic acid (PGA), polyglycolic lactide (PLGA), polyurethane (PU), polylactic acid (PLA), and polycaprolactone (PCL)^8−11^ and also materials extracted from biological resources such as silk fibroin, cellulose-based polymers, alginate, hyaluronic acid, collagen, gelatin, among others have been processed to generate fibrous matrices. These examples are only a part of the versatility of electrospinning when it comes to processing a wide range of polymers. All these mentioned biocompatible membranes have been a proficient tool for the creation of biomedical devices.^12−16^

PCL is a biodegradable material that belongs to synthetic polyester biodegradable polymers such as PGA and PLA. These materials have been approved by the Food and Drug Administration (FDA) to be used in a great variety of fields due to their resistance and long-term degradation.^17^ For example, they are used as main components in sutures, delivery devices, and adhesion barriers.^18^ Some authors suggest the use of PCL in combination with other materials to decrease its hydrophobicity and promote better contact with cells.^19,20^ An adequate material to accomplish this function is collagen. It is a protein with low immunogenicity, present in soft and hard tissues in humans and animals, and plays a main role in maintaining the structure of ECM. The molecular structure of collagen presents the arginine-glycine-aspartic motif that is well known to play a role in cell–cell and cell–substratum interaction.^21−23^ In recent years, there has been a growing interest in alternative sources of collagen due to concerns about animal welfare, ethical considerations, and the desire to diversify sources for sustainability reasons. Some of these alternative sources include marine collagen (derived from fish and seafood byproducts) and collagen from plants, fungi, and bacteria. These alternative sources are being explored as potential substitutes for traditional bovine and porcine collagen in various applications, particularly in response to changing consumer preferences and sustainability goals.^24^ An excellent source of collagen is the skin of tilapia fish; it has noninfectious microbiota, is abundant in type I collagen, and is morphologically similar to the human skin structure.^25,26^ In addition, tilapia fish are cultured locally, and their skin is treated as a waste product, leaving their application as an opportunity area.

The antimicrobial activity of wound dressing is important for diabetic foot ulcer treatment since it is generally infected by *Staphylococcus aureus*, *Enterococcus*, and Gram-negative bacteria such as *Pseudomonas aeruginosa*, *Escherichia coli*, *Klebsiella* species, *Proteus* species, etc.^27^ As most of the wound infections or biofilms are polymicrobial, it is recommended to manage them with wide-spectrum antibiotics. Tetracycline is a favorable drug with antimicrobial properties versus Gram-negative and Gram-positive bacteria, is easily absorbed in all tissues, and has been widely used in the treatment of animal and human infections due to its efficiency, low side effects, and affordable cost.^28^ On the other hand, chloramphenicol is a broad-spectrum antibiotic that exhibits activity against a wide range of bacteria. While it has been associated with various adverse reactions, chloramphenicol has recently gained attention as a potential treatment option for multidrug-resistant Gram-positive bacteria.^29^ As bacteria can rapidly generate antibiotic resistance, this choice of antibiotics can be seen as an option when the microbial consortium does not present resistance.

Based on the aforementioned data and following the interest of previous investigations, this work presents the fabrication and characterization of electrospun blends of PCL and collagen extracted from tilapia skin in combination with tetracycline or chloramphenicol in an attempt to generate nonwoven diabetic foot wound dressings. It is important to note that further research and testing are necessary to validate the efficacy and safety of such wound dressings. Animal studies and eventually clinical trials would be required to assess their performance in diabetic foot ulcer treatments.

## Materials and Methods

### Materials

PCL, *M*_n_: 80,000 from Sigma-Aldrich; 2,2,2-trifluoroethanol, 99.9% (Sigma-Aldrich); rectangular canted neck cell culture flask (Corning); Dulbecco’s modified Eagle’s medium (DMEM, Gibco); fetal bovine serum (Biowest); 1% antibiotic-antimycotic 100× solution (Gibco); tetracycline, 99.9% from Sigma-Aldrich; and chloramphenicol from Sigma-Aldrich were used and also pepsin-soluble collagen, extracted from locally available tilapia using the methodology of González-González et al.^30^

### Membrane Elaboration by the Electrospinning Process

The complete membrane batch was elaborated using an electrospinning technique. This electrospinning set was composed of a SPELLMAN high voltage source (Model CZE 1000R), a KD Scientific syringe power pump (model 2568CO), and a 5 mL solvent-resistant HENKE-JECT syringe with a 21G needle. 2,2,2-Trifluoroethanol was used as the solvent, the concentration of each solution was 10% w/v, and a 10 × 10 × 0.04 cm aluminum plate was used as the collector; during the experiments, relative humidity was 20% and the temperature was 25 °C. The composition and electrospinning parameters applied to each sample are summarized in Table 1. The samples conferred with the name are related to the materials that compose them. Thus, the PCL sample is composed of PCL only, the PCLCOL15 sample is composed of PCL and a 15% in weight of collagen, and the PCLCOL20 sample is composed of PCL and a 20% in weight of collagen. The T or C added to the end of the name indicates the addition of tetracycline or chloramphenicol, respectively. Certainly, the membrane composed of only PCL needed different electrospinning parameters because when using conditions similar to those of the other membranes, the solution tended to drip and the obtained membrane presented beads.

**Table 1:** Composition and Electrospinning Parameters of the Elaborated Membranes

sample	PCL (%)	collagen (%)	tetracycline (%)	chloramphenicol (%)	voltage (kV)	distance (cm)	flow rate (mL/h)
PCL	100				12	18	1.5
PCLCOL15	85	15			16	15	2
PCLCOL20	80	20			16	15	2
PCLCOL15T	85	15	4		16	15	2
PCLCOL20T	80	20	4		16	15	2
PCLCOL15C	85	15		4	16	15	2
PCLCOL20C	80	20		4	16	15	2

### Scanning Electron Microscopy

The microscopic structure of the fibrillar membranes was observed by scanning electron microscopy (SEM) with JEOL, model 5410LV equipment. The samples were cut into 2 × 2 mm squares and glued onto aluminum sample holders using double-sided carbon tape. All of the samples were covered with a sputtered gold film and observed using a 20 kV accelerating voltage. The obtained images were processed with ImageJ software^31^ to calculate the diameter distribution of the fibers, 150 fibers were measured for this purpose.

### Wettability Characteristics by Angle Contact Analysis

The hydrophobicity characteristics of the membrane were measured with a CAM-Plus angle contact meter using a half-angle technique. To carry it out, a drop of deionized water was placed onto the samples using a microsyringe. The measurement was taken a few seconds after the water was dropped.

### Fourier Transform Infrared Spectroscopy

A Frontier PerkinElmer Fourier transform infrared (FTIR)/FIT spectrometer with an attenuated total reflectance (ATR) accessory was used with the purpose of observing the functional groups’ presence and interactions of the different components. The range of measure was 4000–400 cm^–1^ with a 4 cm^–1^ resolution in a transmittance mode.

### Stress–Strain Mechanical Assay

A tensile test was carried out to observe if the properties of the samples accomplish the mechanical characteristics of a wound dressing. The test was performed by using an Instron ElectroPuls System (E1000 Model) with a load cell of 100 N and a deformation rate of 200 mm/min. The samples were cut into 2 cm × 0.5 cm rectangles with an approximate thickness of 0.4 mm.

### Cell Culture and Cellular Viability Test

The cellular line Detroit 548 CCL-116 of human skin fibroblasts was cultured into a 25 cm^2^ rectangular canted neck cell culture flask at 37 °C and 5% of CO_2_ pressure using DMEM supplemented with 10% of fetal bovine serum and 1% of antibiotic-antimycotic 100× solution. Once confluent growth was reached, the cells were detached with 0.125% trypsin–EDTA solution and incubated for 4 min at 37 °C. The obtained cellular suspension was then centrifuged for 4 min at 1000 rpm. The supernatant was decanted, and cells were resuspended in a fresh culture medium. This solution was used to carry out the live/dead staining and viability assay.

To evaluate the cellular viability, the samples were cut into 7 mm diameter discs and sterilized with four cycles of 15 min of UV radiation. After sterilization, the discs were placed at the bottom of the wells of a 96-well plate. Then, an amount of 2500 cells were seeded onto the samples and incubated until subsequent measurements.

The chosen analysis to evaluate the cellular viability was PrestoBlue, a resazurin-based assay, that does not require to solubilize the reaction product and offers comparable results with other commercial assays such as MTT.^32^ The assays were performed after 3, 5, and 7 days of incubation. Once the time was reached, the culture medium was removed and replaced by 200 μL of fresh medium. Subsequently, an amount of 22 μL of PrestoBlue reagent was added to each well and incubated at 37 °C for 4 h in the dark, and then 100 μL of the test medium was aliquoted and analyzed on a 96-well plate. Cells in contact with tissue-cultured plastic (TCP) were used as a positive control.

Absorbance was measured at 570 and 600 nm in a Thermo Scientific Multiskan Sky microplate reader.

Cell viability was obtained from the following equation
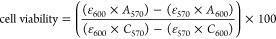
where ε is the molar extinction coefficient of the PrestoBlue reagent, *A* is the absorbance of the sample, and *C* is the absorbance of the cellular control.

### Live/Dead Cellular Stain

Cell behavior on polymeric scaffolds was investigated by a Live/Dead stain assay. 10,000 cells were carefully seeded onto the sample and cultured in a 24-well plate for 3, 5, and 7 days. Once the time was reached, culture media were removed, and the sample was washed three times with PBS (pH = 7.4) solution. After that, 200 μL of a calcein-ethidium homodimer (1:500, 1:1000) solution was added to the well and incubated for 30 min at room temperature. The images were taken using an Eclipse Ti2 fluorescent microscope (Nikon, Ti2A).

### Antimicrobial Activity Test

The antimicrobial activity of electrospun membranes against *S. aureus* (ATCC 6538) and *P. aeruginosa* (ATCC 10,154) was evaluated utilizing the agar diffusion technique based on the experimental section of Vásquez-López.^33^ Both strains were incubated in agar for 24 h at 37 °C before the test. A colony sample of each bacterium was taken from the agar, placed in a test tube with 5 mL of BHI medium, and incubated at 37 °C overnight. The next day, the test tube was centrifuged at 4000 rpm for 15 min. The supernatant was decanted, and the bacteria were resuspended in 1 mL of PBS (pH 7.4) solution. Then, 200 μL of the bacteria suspension was dissolved in 50 mL of PBS solution. The absorbance of the solution was adjusted to 0.1 AU (equivalent to 1 × 10^5^ UFC/mL). The prepared solution was added to 1% BHI–agar medium in a 1:10 proportion, decanted in Petri dishes, and refrigerated at 6 °C. After 30 min of refrigeration, three samples of each membrane with an approximate mass of 0.3 mg were placed on the agar and incubated for 24 h. Subsequently, the inhibition zone of each sample was measured with the software ImageJ using a ruler as a length reference.

## Results and Discussion

### Morphological Analysis by SEM

**Figure 1:**
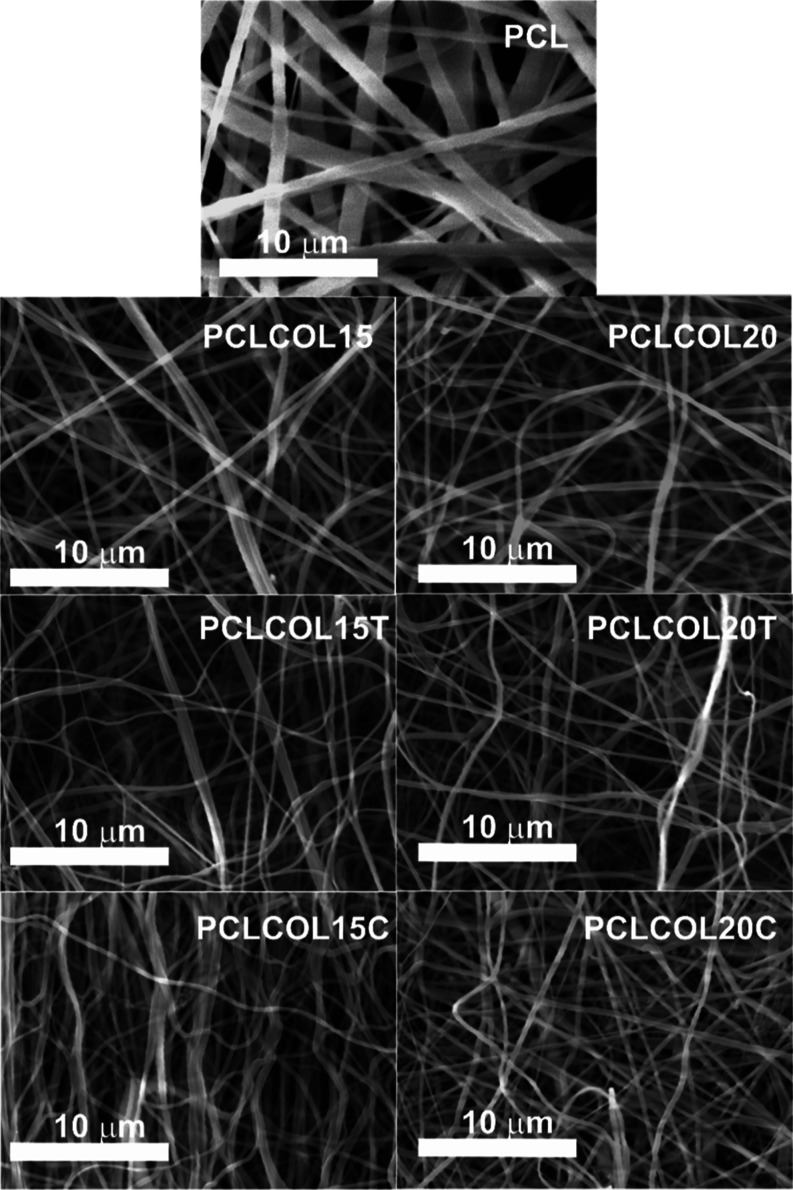
SEM at 5000× of the PCL, PCLCOL15, PCLCOL20, PCLCOL15T, PCLCOL20T, PCLCOL15C, and PCLCOL20C samples.

Figure 1 shows the SEM micrographs of all of the electrospun membranes. PCL sample is a membrane composed of ribbons with higher dimensions than the other materials. The rest of the samples presented a fibrous shape and a tendency of the fibers to merge side by side; this is observed in all the samples with collagen and PCL, especially in the PCLCOLC15 sample. The samples’ morphology presented ribbons and fibers with a smooth profile that can topographically mimic the extracellular matrix which means an approach to function as a tissue scaffold.

**Figure 2:**
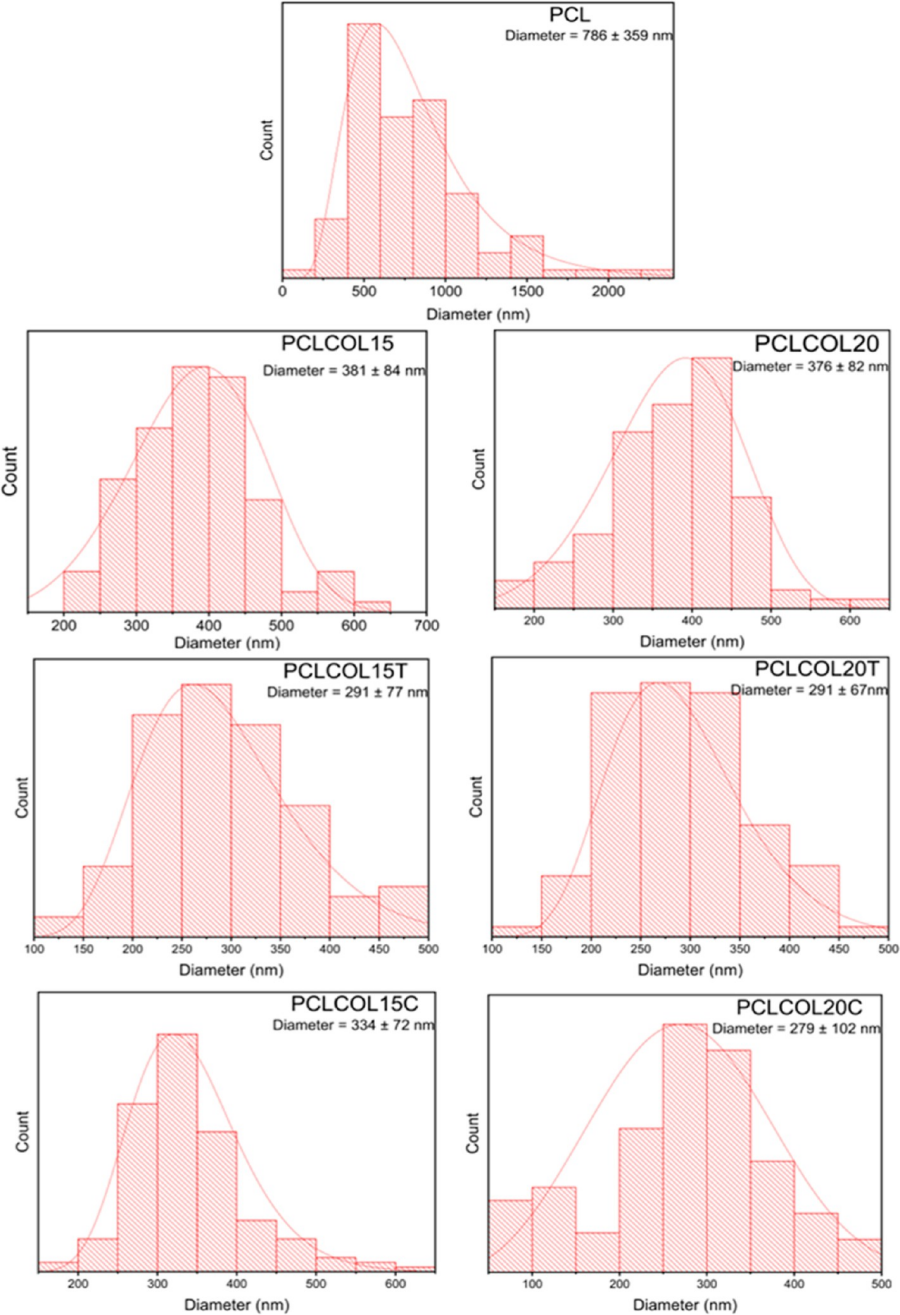
Fiber diameter distribution of the PCL, PCLCOL15, PCLCOL20, PCLCOL15T, PCLCOL20T, PCLCOL15C, and PCLCOL20C samples.

The fiber diameter distribution and the average diameter of each membrane are presented in Figure 2. PCL sample presented the highest average, about 786 ± 359 nm. Lower measurements were observed for the PCLCOL15 and PCLCOL20 samples which exhibit diameters of 380 ± 84 and 376 ± 82 nm, respectively. This average diameter decreased even more when tetracycline and chloramphenicol were included in the membranes’ composition. For PCLCOL15T and PCLCOL20T, these values were 291 ± 76 and 291 ± 67 nm, respectively. In the case of membranes with chloramphenicol, the average diameters were 334 ± 72 and 279 ± 102 for PCLCOL15C and PCLCOL20C, respectively. The electrospinning of PCL is improved when it is mixed with other polymers and molecules; this effect has been observed in other investigations also;^34^ this correlates with the decrease in fiber diameter when collagen and antibiotics are included.

### Wettability Characteristics by Angle Contact Analysis

Table 2 shows the water contact angle obtained by the half-angle technique. The PCL membrane shows a contact angle of 148 ± 10° and can be considered as a hydrophobic material since this value is above 90°.^35^ The fibrous samples that contain tilapia collagen show a contact angle of around 60° for those with 15% collagen and lower values for those which contain 20% collagen in their composition. All of the composite materials presented hydrophilic characteristics. The PCLCOL20C sample was unmeasurable since the drop was quickly absorbed by the sample, making the measurement impossible.

**Table 2:** Water Contact Angle of the Electrospun Membranes

sample	water contact angle (deg)
PCL	148 ± 10
PCLCOL15	61 ± 13
PCLCOL20	54 ± 2
PCLCOL15T	60 ± 5
PCLCOL20T	47 ± 4
PCLCOL15C	59 ± 2
PCLCOL20C	ND

### Chemical Analysis Using FT-IR Spectroscopy

**Figure 3:**
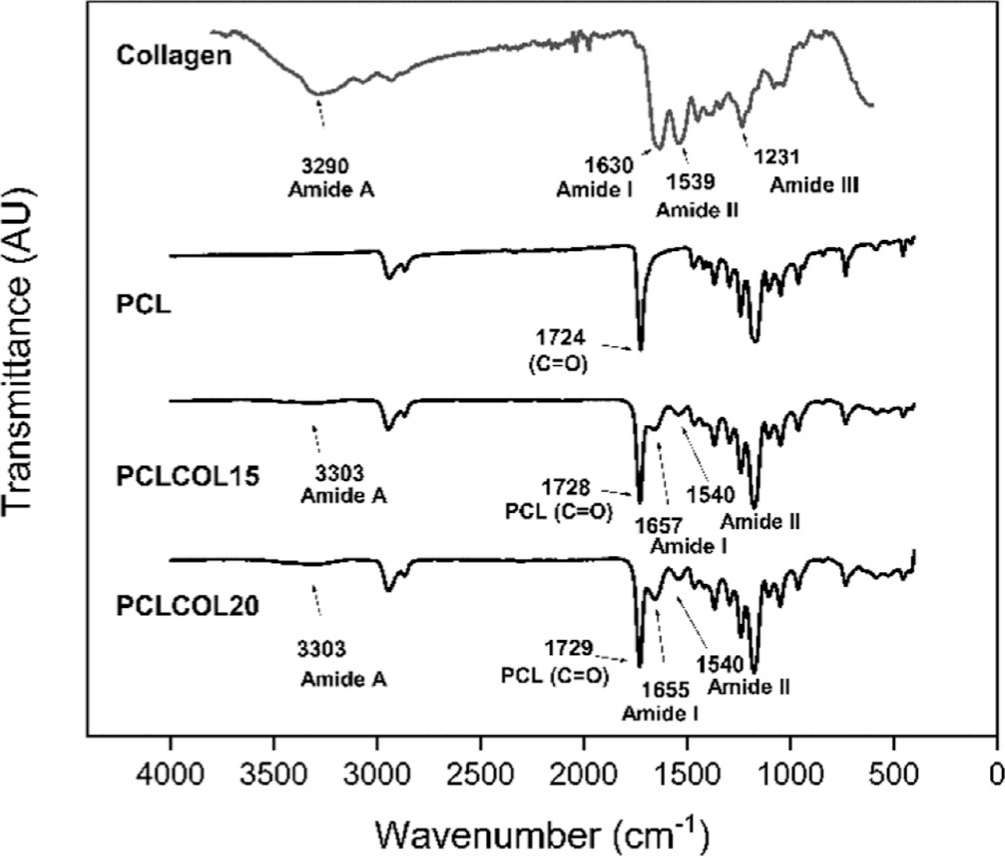
ATR-FTIR spectra of the pepsin soluble collagen (used as raw material in the membrane elaboration) and PCL, PCLCOL15, and PCLCOL20 membranes.

FTIR spectra of collagen, PCL, PCLCOL15, and PCLCOL20 samples are shown in Figure 3. The collagen membrane spectra exhibit the vibrations of its structural amide bond, which are classified as amides A, B, I, II, and III. The amide A band corresponds to the vibrational stretching of the (N–H) bond and it appears at 3290 cm^–1^ and the amide B signal is the second component of the amide A Fermi resonance that weakly appears between 3100 and 3030 cm^–1^. The amide I band is principally generated by the carbonyl (C=O) stretching vibration of the peptide group and can be seen at 1630 cm^–1^. The amide II signal is composed of (N–H) bending and (C–N) stretching vibrations and is found at 1539 cm^–1^. Finally, the amide III with contributions of (N–H) bending and (C–N) stretching vibrations is observed at 1231 cm^–1^.^36^ The PCL sample shows the characteristic bands of the functional groups of its backbone structure. The bands for the asymmetric and symmetric stretching of the CH_2_ group can be seen at 2941 and 2864 cm^–1^, respectively. Also, a sharp and intense band at 1724 cm^–1^ for carbonyl stretching (C=O) is observed. Finally, the asymmetric and symmetric bands for the C–O–C bonds can be noticed at 1240 and 1166 cm^–1^, respectively.^37^ In the PCLCOL15 and PCLCOL20 spectra, the main bands of both collagen and PCL are presented, and the amide A (N–H, 3290 cm^–1^) and amide I (C=O, 1630 cm^–1^) of collagen and the carbonyl group (C=O, 1724 cm^–1^) of PCL are shifted to higher energy, indicating a hydrogen bond interaction between both materials. In addition, a slight increment in the intensity of amide I and amide II can be observed in PCLCOL20 compared to PCLCOL15.

**Figure 4:**
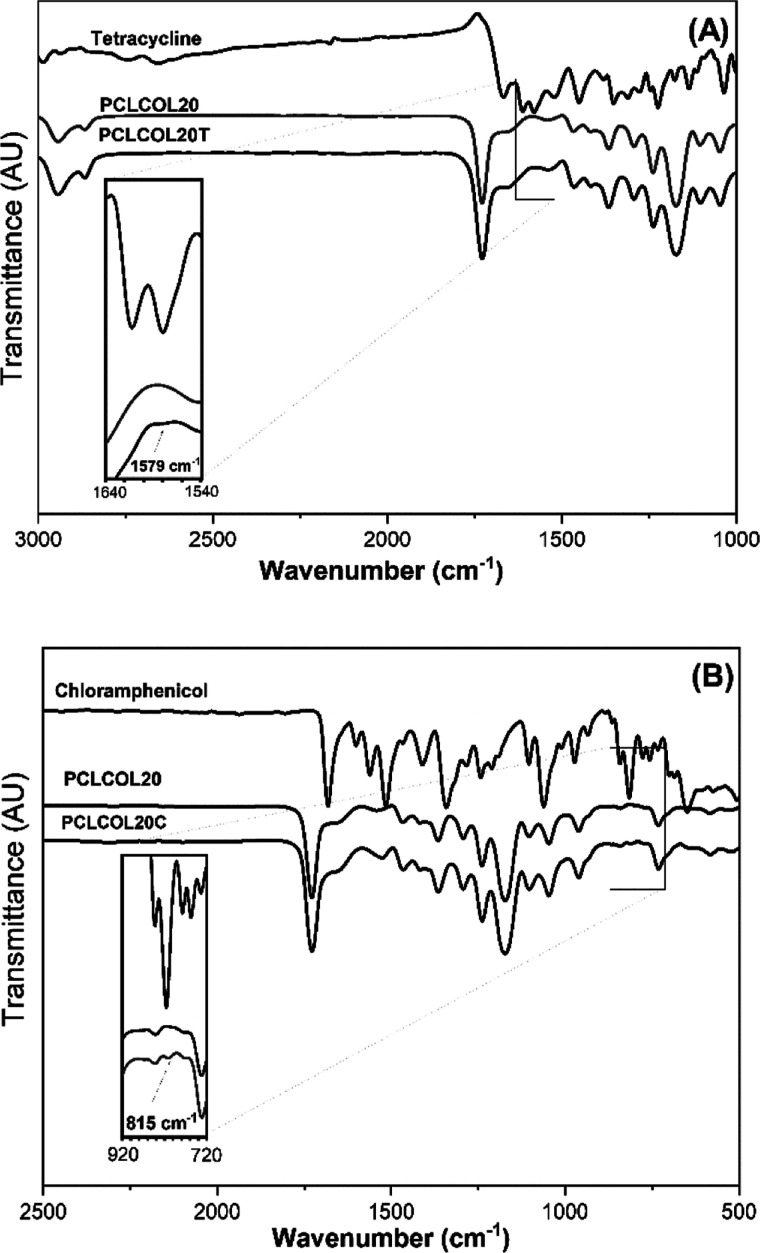
(A) ATR-FTIR spectra of tetracycline and PCLCOL20 and PCLCOL20T membranes. (B) ATR-FTIR spectra of chloramphenicol and PCLCOL20 and PCLCOL20C membranes.

Figure 4 section (A) shows the FTIR spectra of tetracycline, PCLCOL20 sample, PCLCOL20T sample, and a chart showing a magnification between 1540 and 1640 cm^–1^. In the chart, there is evidence of the presence of tetracycline in the PCLCOL20T sample due to the existence of a band at 1579 cm^–1^ corresponding to C=C stretching vibration of tetracycline;^38^ this signal is not seen in the spectra of PCLCOL20 sample. Analogous behavior is presented in section B of Figure 4, showing the FTIR spectra of chloramphenicol, PCLCOL20 sample, PCLCOL20C sample, and a magnification of them, where a band, tentatively assigned to out-of-plane vibrations of the ortho-disubstituted aromatic ring of chloramphenicol, can be observed at 815 cm^–1^ in the PCLCOL20C sample,^39^ while it does not appear in the PCLCOL20 sample, indicating the presence of chloramphenicol in the PCLCOL20C sample.

### Mechanical Analysis of the Membranes under Tensile Stress

**Figure 5:**
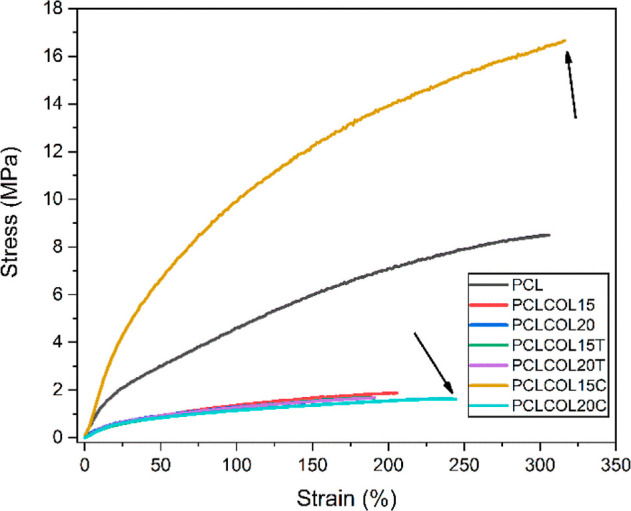
Stress–strain curve of the PCL, PCLCOL15, PCLCOL20, PCLCOL15T, PCLCOL15C, and PCLCOL20C membranes.

The elastic modulus, yield point, and tensile strength obtained for the membranes are summarized in Table 3. PCL sample presents mechanical properties about 4 times higher than those composed of collagen, PCL, and drug, except for the PCLCOL15C sample; in this particular case, an increase in all the mechanical properties was observed (almost double values for elastic modulus and tensile strength and 3 times for yield point). The higher mechanical properties of PCL correlate to the higher width of the fibers that compose it. The behavior presented by PCLCOL15C can be justified by the tendency of the fibers to merge side by side as seen in SEM analysis. Figure 5 shows the stress/strain plot of all of the samples where the mentioned behavior and also an increase in strain to fail in the samples containing chloramphenicol can be seen. The samples composed of the blend of PCL and collagen showed an elongation to failure of 186 and 156% for PCLCOL15 and PCLCOL20, respectively. Membranes containing tetracycline showed a tendency to failure around a strain of 195%, while the values for samples containing chloramphenicol increased to 247 and 320% for PCLCOL20C and PCLCOL15C, respectively (marked with an arrow).

**Table 3:** Mechanical Properties of the Elaborated Membranes

	modulus (kPa)	yield (kPa)	tensile strength (kPa)	elongation to failure (%)
PCL	115.48 ± 4.5	1167 ± 78.6	8586 ± 325	308 ± 3
PCLCOL15	34.6 ± 4.0	250 ± 12.1	1990 ± 104	186 ± 21
PCLCOL20	25.9 ± 4.3	211 ± 20	1363 ± 151	156 ± 7
PCLCOL15T	35.5 ± 1.7	245 ± 13.7	1645 ± 94	201 ± 8
PCLCOL20T	33.9 ± 6.7	238 ± 2.5	1515 ± 21	190 ± 8
PCLCOL15C	231.2 ± 34	3453 ± 83	17162 ± 2270	320 ± 1
PCLCOL20C	33.5 ± 5.6	378 ± 19.7	1727 ± 77	247 ± 3

#### Cellular Viability Test

**Figure 6:**
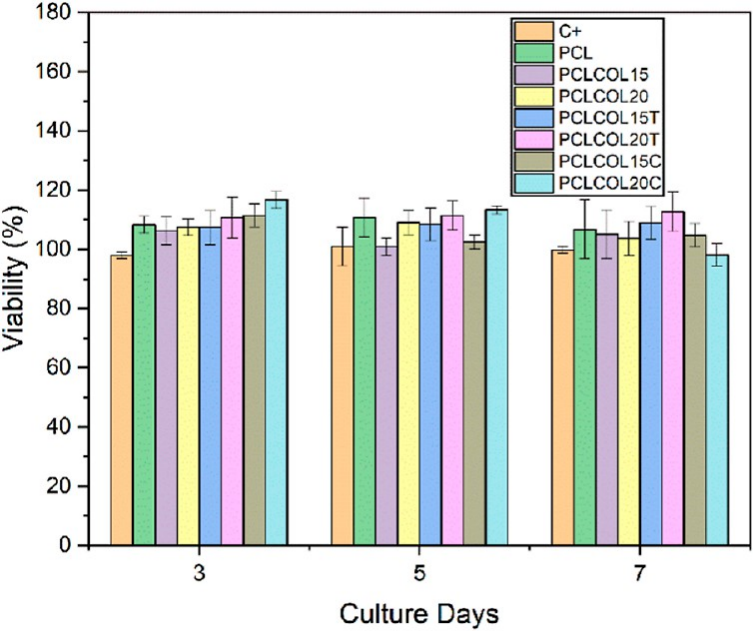
Bar plot of cellular viability of Detroit 548 CCL 116 cell line in contact with the PCL, PCLCOL15, PCLCOL20, PCLCOL15T, PCLCOL20T, PCLCOL15C, and PCLCOL20C samples. The TCP was used as a control.

The cellular viability assay was performed using PrestoBlue (resazurin) reagent. Each material was subjected to this test in order to prove if the membranes can function as a cellular host and to assess if the presence of tetracycline and chloramphenicol disturbs the cellular behavior. Figure 6 shows the viability of the cellular line Detroit 548 CCL-116 of human skin fibroblasts after being in contact with the samples for a period of 3, 5, and 7 days. The results showed good cellular behavior. Almost all the samples presented superior cell viability than those in contact with tissue culture polystyrene after 3, 5, and 7 days. The only exception happened on day 7 where the cells in contact with PCLCOL20C presented a cell viability of 98%, nevertheless, it can be considered as a non cell-disturbing material.

#### Live/Dead Assay

**Figure 7:**
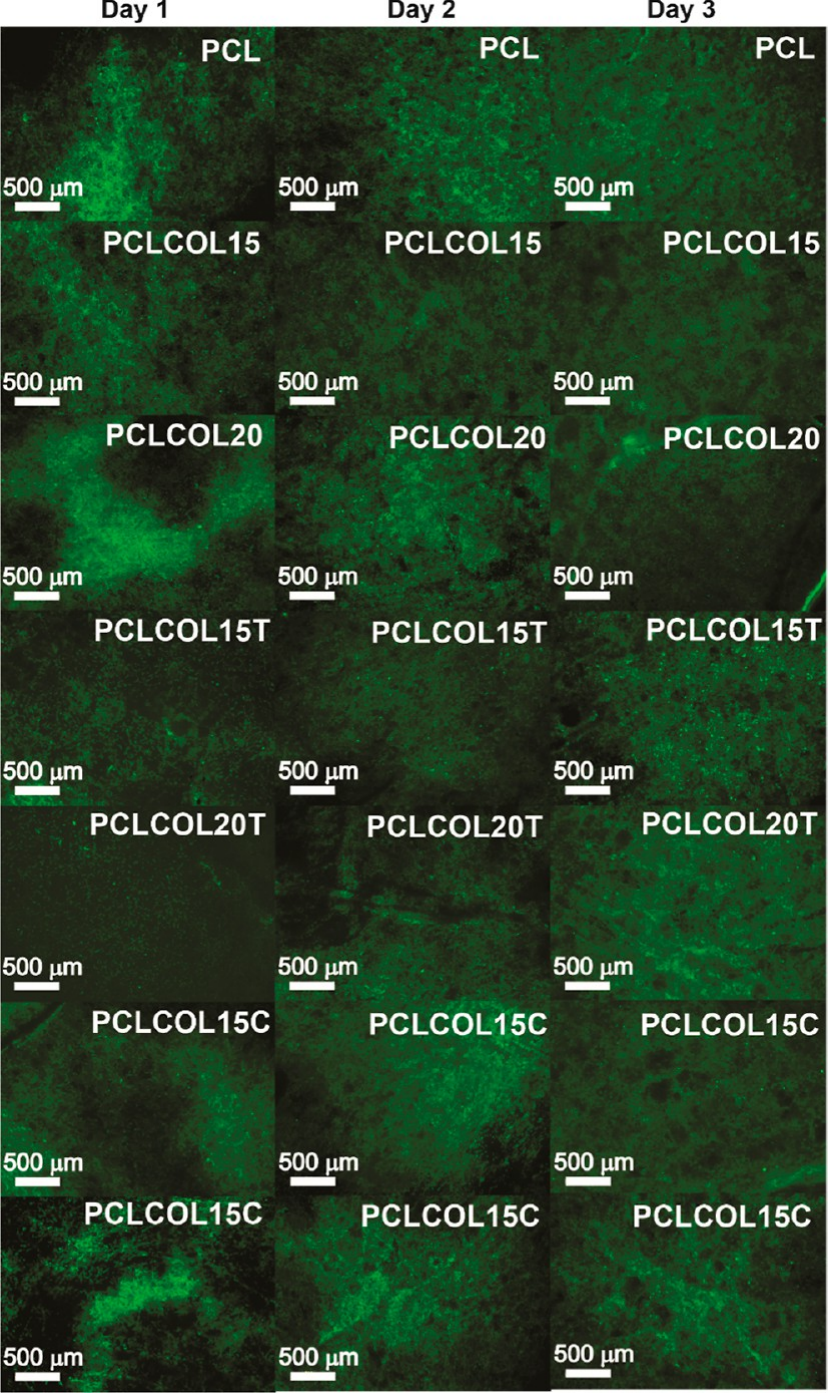
Live/dead cell double staining with calcein-ethidium homodimer-1 of Detroit 548 CCL 116 human fibroblast cell line after being in contact for 3, 5, and 7 days with the PCL, PCLCOL15, PCLCOL20, PCLCOL15T, PCLCOL20T, PCLCOL15C, and PCLCOL20C samples.

**Figure 8:**
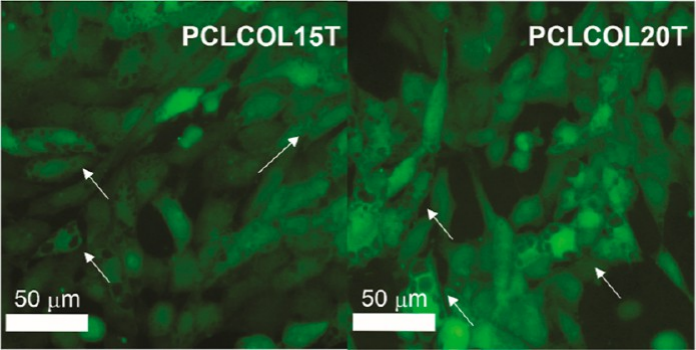
Live/dead cell stain with calcein-ethidium homodimer of Detroit 548 CCL 116 human fibroblast cell line after being in contact for 3 days with the PCLCOL15T and PCLCOL20T samples.

Figure 7 shows the live/dead cell double staining with calcein-ethidium homodimer-1 of Detroit 548 CCL 116 human fibroblast cell line after being in contact for 3, 5, and 7 days with the PCL, PCLCOL15, PCLCOL20, PCLCOL15T, PCLCOL20T, PCLCOL15C, and PCLCOL20C samples. Dead cells (red) were not observed, even after 7 days of exposure in all the samples; this indicates that the material is compatible with cells and suggests that they can be used as wound dressings or scaffolds. However, the cells in contact with the membranes containing tetracycline (PCLCOL15T and PCLCOL20T) presented vesicles, revealing that the tetracycline altered the cellular metabolism, as can be seen in Figure 8. This behavior can be related to lipid accumulation in response to the presence of tetracycline.^40^

### Antibacterial Activity

**Figure 9:**
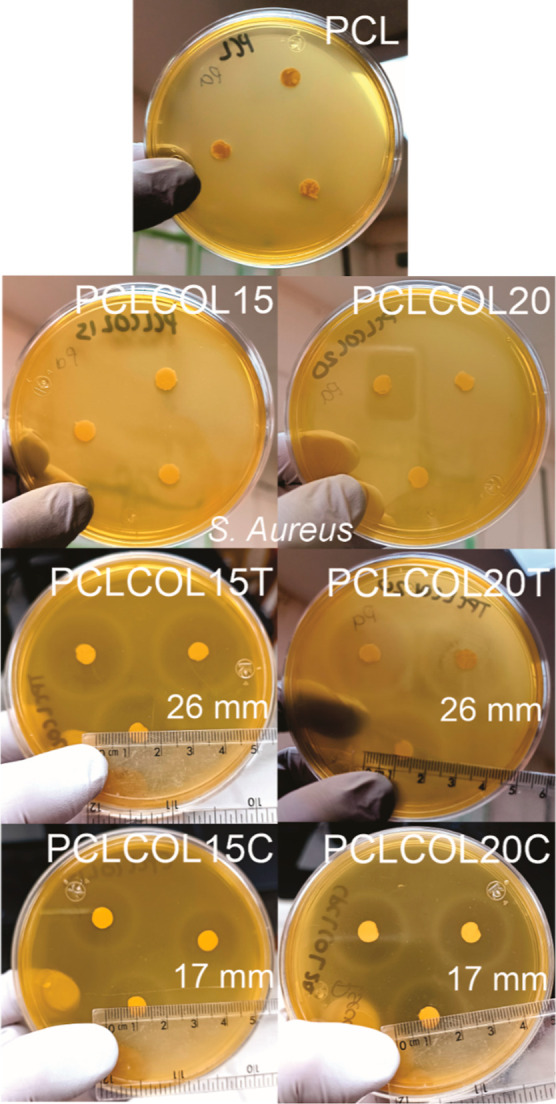
*S. aureus* inhibition for electrospun membranes at 24 h of PCL, PCLCOL15, PCLCOL20, PCLCOL15T, PCLCOL20T, PCLCOL15C, and PCLCOL20C samples’ exposition.

**Figure 10:**
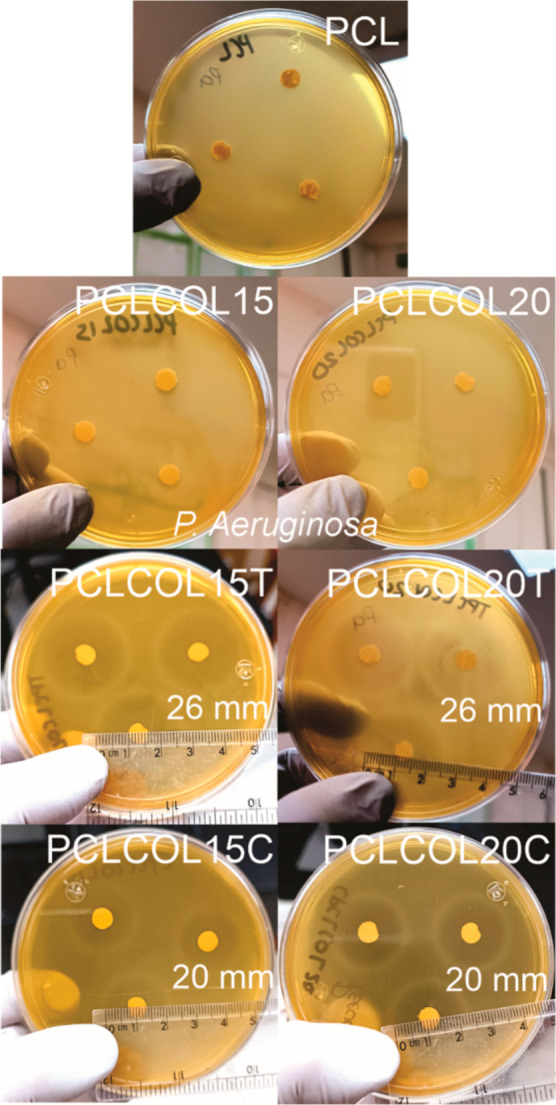
*P. aeruginosa* inhibition for electrospun membranes at 24 h of PCL, PCLCOL15, PCLCOL20, PCLCOL15T, PCLCOL20T, PCLCOL15C, and PCLCOL20C samples’ exposition.

Figures 9 and 10 show the antibacterial effects of tetracycline and chloramphenicol against *S. aureus* (ATCC 6538) and *P. aeruginosa* (ATCC 10,154) cultured on 1% BHI-agar. The lack of inhibition halo around the rounded PCL, PCLCOL15, and PCLCOL20 samples showed that there is no antimicrobial effect against both strains.

In the *S. aureus* case (Figure 9), the sample discs PCLCOL15T and PCLCOL20T showed an inhibition halo of a 26 mm diameter, which is considered as proof of antibiotic sensitivity. For the PCLCOL15C and PCLCOL20C samples, the diameter of the inhibition halo was 17 mm; in this case, the inhibition halo demonstrates a less effective antimicrobial activity^41^ as compared to membranes with tetracycline.

Regarding the activity against *P. aeruginosa* (Figure 10), this strain was shown to be sensitive to PCLCOL15T, PCLCOL20T, PCLCOL15C, and PCLCOL20C samples. An inhibition halo greater than 19 mm for tetracycline and 18 mm for chloramphenicol is considered as proof of sensitivity.^41^

## Conclusions

This study describes the elaboration and characterization of electrospun membranes composed of a combination of PCL and collagen extracted from tilapia skin. Tetracycline and chloramphenicol were also added to the membranes. SEM analysis revealed that the PCL membrane consisted of ribbons, while the combined PCL and collagen membranes (PCLCOL15, PCLCOL20, PCLCOL15T, PCLCOL20T, PCLCOL15C, and PCLCOL20C) were composed of smooth fibers. The fibers of the PCLCOL15C sample showed a tendency to merge side by side, which resulted in an increase in mechanical properties. FTIR spectroscopy confirmed the presence of the components in the membranes and suggested an interaction through hydrogen bonding, as evidenced by a shift in the principal functional group in the backbone structures of the polymeric components. Cell viability tests showed values over 100% after 3, 5, and 7 days for all of the materials, indicating that the materials did not disrupt cellular redox activity. Dead cells were not observed in the live/dead staining test, and cells spread out above all of the membranes, indicating favorable cell compatibility. Cells in contact with the samples containing tetracycline exhibited vesicles, indicating a change in cellular metabolism similar to lipid accumulation in response to the presence of tetracycline. The samples containing tetracycline or chloramphenicol exhibited antimicrobial activity against *S. aureus* (ATCC 6538) and *P. aeruginosa* (ATCC 10,154) strains. It is important to note that diabetic foot infections are polymicrobial and hard to treat. In this case, PCL, collagen, tetracycline, and chloramphenicol were presented as electrospun materials with satisfactory cellular hosting and antibacterial behavior. These findings suggest that the developed materials have the potential to be further explored as wound dressings or scaffolds for diabetic foot ulcers. The next step in the research is to evaluate the performance of these materials in vivo and explore more compositions, components, and antimicrobial agents. Taking into account that the goal is to ensure that these systems are safe, effective, and capable of reducing the misuse and abuse of antibiotics, ultimately helping to combat drug resistance and improve patient outcomes.
